# SARS-CoV-2 Omicron Variant in Human Saliva Samples in Cell-Free Form

**DOI:** 10.1001/jamanetworkopen.2022.50207

**Published:** 2023-01-09

**Authors:** Kenichi Imai, Ryo Ikeno, Hajime Tanaka, Norio Takada

**Affiliations:** 1 Department of Microbiology and Immunology, Nihon University School of Dentistry, Tokyo, Japan; 2 Asuyoshi Dental Clinic, Yokohama, Japan; 3 Takeda’s Medical Office of Internal Medicine, Nagoya, Japan

## Abstract

This cross-sectional study investigates cell-free viral loads in saliva samples from patients who have been infected with the Omicron variant of SARS-CoV-2.

## Introduction

Infection with SARS-CoV-2 can be measured in saliva samples as well as nasopharyngeal swabs^1^; specimens contain infectious virions released from the oral epithelia and salivary glands,^2^ either in association with detached oral epithelial cells or in cell-free form. However, the relative contribution of cell-free virions in viral transmission has received little attention. Recently, an increase in secondary household infections has been observed amidst the Omicron variant surge,^3^ possibly due to increased aerosol transmission. We investigated cell-free viral loads in saliva samples from patients infected with SARS-CoV-2.

## Methods

This cross-sectional study followed the STROBE reporting guideline and was approved by the Ethics Committee of Nihon University School of Dentistry. Study participants were Japanese patients who randomly came to an outpatient clinic in Nagoya, Japan, and were determined to be SARS-CoV-2 positive. They were given oral and written explanations in advance and notified of their right to withdraw from the study.

Whole saliva specimens were collected from patients infected either with a wild-type strain (WT), the Delta variant, or the Omicron variant (Table). Viral RNA extraction and real-time quantitative polymerase chain reaction were conducted according to the National Institute of Infectious Diseases of Japan protocols.^4^ Synthetized viral RNA was used to construct a standard using Poisson null distribution. Cell-free supernatants were generated by centrifugation (10 000*g* for 5 minutes) of whole saliva samples and contained only 0.5% host DNA, which was determined using human chromosome-specific quantitative polymerase chain reaction.

**Table.** Participant Characteristics

Characteristic	Strain			*P* value
	Wild-type strain (n = 22)^a^	Delta variant (n = 32)^b^	Omicron variant (n = 36)^c^	
Sex, No.				
Male	12	20	17	.45^d^
Female	10	12	19	
Age, mean (SD), y	39.5 (18.6)	32.4 (14.8)	38.4 (16.3)	.20^e^
Days since onset, mean (SD)	2.6 (1.4)	3.6 (2.6)	2.1 (1.4)	.009^e^

^a^ Collected in November and December 2020.

^b^ L452R mutation collected in August and September 2021.

^c^ G339D mutation collected in January and February 2022.

^d^ Examined using the χ^2^ test.

^e^ Examined using analysis of variance.

Percentage sex distribution was assessed using the χ^2^ test, and mean (SD) age and number of days since onset were assessed using analysis of variance. Viral copy numbers in whole saliva samples and centrifuge supernatant and the ratio of viral load in the supernatant to that in the whole saliva samples were compared among the 3 strains using the Dunn-Bonferroni test. Analyses used SPSS Statistics software, version 28,0.1.1(14) (IBM Corporation), and 2-sided *P* < .05 indicated statistical significance.

## Results

Characteristics of the 90 participants (49 men and 41 women; mean [SD] age, 36.5 [16.5] years) are provided in the Table. In whole saliva samples, the median viral load was 15.1-fold higher in Delta than WT strains but only 7.7-fold higher in Omicron than WT strains (Figure, A). Viral load in the cell-free supernatants was only 6.5-fold higher in Delta than WT strains, which was not significant. Conversely, viral load in the Omicron samples was 17.7-fold higher than in WT samples (Figure, B). Omicron samples displayed higher ratios of viral load in the supernatant vs that in whole saliva compared with the WT and Delta strains (Figure, C).

**Figure.**
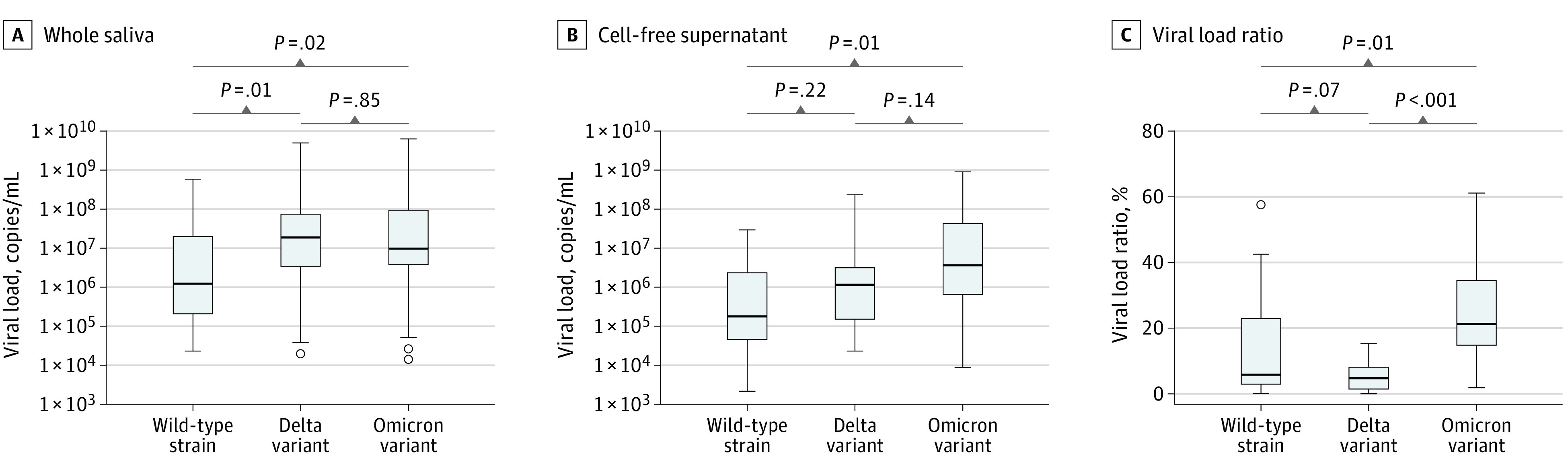
Comparisons of Viral Copy Number and Viral Load Ratio Comparisons of the median (IQR) viral copy number in whole saliva samples (A), its cell-free supernatant (B), and viral load ratio as the percent ratio of the viral copy number in the whole saliva sample and its cell-free supernatant (C) among the wild-type, Delta, and Omicron variant samples (examined using Dunn-Bonferroni test).

## Discussion

Droplets must be smaller than 5 μm in diameter to be dispersed by aerosol; human epithelial cells are larger than 10 μm in diameter. Thus, viruses that spread through aerosol transmission are likely to exist in cell-free form. Even if detached virus-associated epithelial cells could be transmitted via aerosol droplets, virions that are newly released from host cells take 2 to 3 times longer to infect a new host cell compared with cell-free virus, even after repeated passages.^2^ Therefore, the propensity of a virus to be excreted from host cells in high levels is essential to its ability to be transmitted via aerosol. In addition, the Omicron variant has a 2- to 5-fold higher growth rate in the human population^5^ and a higher binding to angiotensin-converting enzyme 2^6^ compared with the Delta variant. These findings, together with our finding that the Omicron variant was more prevalent in the cell-free form in saliva samples, suggest greater potential for aerosol transmissibility. This could explain the increased secondary household infection rate.^3^

A limitation of this study was that it did not examine whether the cell-free Omicron variant in saliva samples contributed to aerosol infections. Routine attention should be given to the shedding of cell-free form of progeny viruses into saliva.

## Notes

Supplement.
Data Sharing Statement
