# Supplementary material for: SARS-CoV-2 Omicron Variant in Human Saliva Samples in Cell-Free Form
Source: JAMA Netw Open. 2023 Jan 9;6(1):e2250207. doi: 10.1001/jamanetworkopen.2022.50207 (PMC9857355; doi:10.1001/jamanetworkopen.2022.50207)
Supplement: Supplement. — Data Sharing Statement [file jamanetwopen-e2250207-s001.pdf]

Data Sharing Statement

Imai. SARS-CoV-2 Omicron Variant in Human Saliva Samples in Cell-Free Form. *JAMA Netw Open*. Published January 09, 2023. doi:10.1001/jamanetworkopen.2022.50207

Data
Data available: Yes
Data types: Participant data with identifiers
How to access data: imai.kenichi@nihon-u.ac.jp
When available: With publication

Supporting Documents
Document types: None

Additional Information
Who can access the data: researchers whose proposed use of the data has been approved
Types of analyses: for a specified purpose
Mechanisms of data availability: after approval of a proposal
